# Supplementary material for: MetaRibo-Seq measures translation in microbiomes
Source: Nat Commun. 2020 Jun 29;11:3268. doi: 10.1038/s41467-020-17081-z (PMC7324362; doi:10.1038/s41467-020-17081-z)
Supplement: Supplementary file 10 — Supplementary Data 7 [file 41467_2020_17081_MOESM10_ESM.zip › File2/Confidence_High_Taxonomy/307343_out.krona.html]

Javascript must be enabled to view this page.

members
magnitude
magnitudeUnassigned
count
unassigned
taxon
rank

307343\_out

9

9
superkingdom
2

976
phylum
9

200643
class
9

order
9
171549

815
family
9

9
genus
816


SRS1041039\_contig\_number\_78
species
1
47678

162156
species
1

SRS140492\_contig\_number\_6365

1739298

SRS103987\_contig\_number\_26129
species
1

1263044

SRS148319\_contig\_number\_4022
1
species

species
2

SRS014287\_contig\_number\_11451SRS971276\_contig\_number\_23839
818

674529

SRS018623\_contig\_number\_38SRS146764\_contig\_number\_4017SRS148159\_contig\_number\_33998
species
3
